# Integrated Analysis of Human Hemorrhagic Fever with Renal Syndrome Cases and Rodent Hantavirus Surveillance in Zhejiang Province, China, 2025

**DOI:** 10.3390/v18090945

**Published:** 2026-08-28

**Authors:** Rong Zhang, Qian-Hui Zheng, Haizhen Zhou, Fang Xu, Yi-Sheng Sun, Ji-Min Sun, Li-Qun Fang, Li-Ping Wang

**Affiliations:** 1 National Key Laboratory of Intelligent Tracking and Forecasting for Infectious Diseases, Division of Infectious Disease, Chinese Center for Disease Control and Prevention & Chinese Academy of Preventive Medicine, No. 155, Chang bai Road, Changping District, Beijing 102206, China; rzhang@cdc.zj.cn; 2 Zhejiang Key Lab of Vaccine, Infectious Disease Prevention and Control, Zhejiang Provincial Center for Disease Control and Prevention, No. 3399, Binsheng Road, Binjiang District, Hangzhou 310051, China; 3 Kaihua Center for Disease Control and Prevention, Quzhou 323000, China; 4 Wencheng Center for Disease Control and Prevention, Wenzhou 325300, China; 5 State Key Laboratory of Pathogen and Biosecurity, Academy of Military Medical Science, No. 20, Dongdajie, Fengtai District, Beijing 100071, China

**Keywords:** hantavirus, rodent surveillance, One Health, reservoir hosts, hemorrhagic fever with renal syndrome

## Abstract

Hemorrhagic fever with renal syndrome (HFRS), caused by Seoul orthohantavirus (SEOV) and Hantaan orthohantavirus (HTNV), remains a neglected rodent-borne zoonosis with persistent residual spillover risk in low-incidence regions of China. This study assessed human HFRS occurrence and small-mammal hantavirus surveillance signals in small-mammal hosts across Zhejiang Province, China, in 2025. Reported human cases were obtained from the National Notifiable Disease Surveillance System, and animal host surveillance was conducted at five designated monitoring sites. A total of 107 HFRS cases were reported, occurring year-round with peaks in June and December and showing spatial heterogeneity, presenting a predominance of males, adults aged ≥40 years, and farmers. Animal host surveillance captured 617 small mammals (566 rodents and 51 shrews) from 15,457 valid trap-nights, with *Apodemus agrarius* (30.31%) and *Rattus norvegicus* (29.98%) as the dominant species. Sixteen animals were positive for hantavirus RNA and/or antibody, including 12 for hantavirus RNA and 6 antibody-positive animals; two were positive for both markers. Positive animals were detected in multiple species, including *Niviventer confucianus*, *R. norvegicus*, *Rattus losea*, *A. agrarius*, *Suncus murinus*, and *Rattus tanezumi*. Human incidence, small-mammal density, host species composition, and hantavirus-positive rates were not fully concordant across surveillance sites. Our findings reveal sustained cryptic orthohantavirus circulation in low-endemic Zhejiang, highlighting that integrated human-reservoir One Health surveillance is indispensable for precise spillover risk prediction and targeted zoonotic disease intervention.

## 1. Introduction

Hemorrhagic fever with renal syndrome (HFRS) is a rodent-borne zoonotic disease caused by hantaviruses and remains an important public health concern in many parts of Eurasia, particularly in China [1,2]. Humans are usually infected through inhalation of aerosols or contact with environments contaminated by the urine, feces, or saliva of infected rodents [3]. The clinical spectrum of HFRS ranges from mild febrile illness to severe disease characterized by fever, hemorrhagic manifestations, thrombocytopenia, increased vascular permeability, hypotension, and acute kidney injury [4,5]. In China, HFRS is primarily caused by two hantavirus species: Hantaan virus (HTNV) and Seoul virus (SEOV). HTNV infection is generally associated with more severe clinical manifestations and higher case fatality rates, which may reach approximately 5–10% in severe endemic settings, whereas SEOV infection is usually milder with lower fatality rates (approximately 1%). Although all populations are susceptible to hantavirus infection, the risk of infection is largely determined by exposure opportunities, particularly contact with rodent reservoirs and contaminated environments. Historically, HFRS has predominantly affected rural populations, especially farmers and individuals engaged in agricultural activities, due to their frequent interactions with rodent habitats [5]. Because early symptoms may overlap with those of other acute febrile illnesses, timely clinical recognition and laboratory confirmation are essential for patient management, surveillance sensitivity, and targeted outbreak response [2].

China has historically reported a substantial burden of HFRS, and hantavirus transmission remains heterogeneous across ecological and geographic settings. Recent national surveillance indicates that HFRS continues to occur in China, with the distribution and diversity of hantaviruses influenced by ecological environment, climate, host distribution, and social development. National surveillance from 2014 to 2023 revealed that although HFRS incidence declined from 1.01 to <0.4 per 100,000, the affected geographic area expanded to 22 provincial regions, with 12 Hantaan virus genotypes and 9 Seoul virus genotypes circulating. Active viral transmission in rodent populations persists even in areas with few or no human cases, underscoring a continued risk of epidemic resurgence [6]. In the broader context of vector-borne diseases in China, HFRS remains a major component. From 2005 to 2024, a total of 1,129,736 vector-borne disease (VBD) cases were reported nationwide, among which HFRS accounted for 18.4% of all VBD cases, ranking third after scrub typhus (28.17%) and malaria (20.8%) [7].

Zhejiang Province, located in eastern China, is a recognized natural focus of HFRS. Long-term monitoring data from 1963 to 2020 show that although the overall incidence rate has significantly decreased since the peak in the 1980s, the disease has consistently shown a marked male predominance and a high concentration in the farming population (approximately 78%). Moreover, in recent decades, the age distribution of cases has gradually shifted towards the elderly, and the geographical range of the epidemic area has expanded eastward, indicating that ecological conditions have continued to change, maintaining the transmission of Hantavirus [8]. Long-term surveillance has shown that HFRS incidence in Zhejiang has declined markedly compared with historical high-incidence periods, likely reflecting improvements in rural environments, rodent control, vaccination, public health surveillance, and clinical diagnosis [9]. However, sporadic human cases and localized endemic foci continue to be reported, suggesting that enzootic hantavirus circulation persists in Zhejiang Province despite the substantial reduction in disease incidence [10]. Under a low-incidence setting, the spatial heterogeneity of human cases may become more apparent, and sporadic infections in non-high-burden areas may be easily overlooked, potentially leading to delayed diagnosis. HFRS shares overlapping clinical manifestations with other endemic febrile diseases (such as Severe Fever with Thrombocytopenia Syndrome (SFTS)), which further increases the difficulty of differential diagnosis. Although both of these diseases have rural epidemic characteristics and similar early symptoms, they exhibit different epidemiological features in terms of age and gender distribution, highlighting the importance of differential diagnosis and timely laboratory confirmation in clinical practice, especially in areas with a lower incidence rate. Local awareness of HFRS may be insufficient in such regions [11].

Zhejiang Province is located in southeastern China and has a warm and humid subtropical monsoon climate, with diverse landscapes including plains, hills, mountainous areas, and rural agricultural environments. However, changes in land use, population mobility, and human–environment interactions have resulted in more complex exposure patterns beyond traditional rural settings. These ecological conditions provide suitable habitats and resources for rodent hosts, while agricultural activities and frequent human–rodent interactions in rural areas may increase the opportunities for hantavirus exposure. HFRS is a typical One Health disease driven by the complex interactions among humans, rodent reservoirs, pathogens, and environmental factors. Although long-term integrated surveillance, environmental improvement, and sustainable rodent management strategies have likely contributed to the continuous decline of HFRS incidence in Zhejiang Province, the persistence of natural reservoir habitats means that the risk of hantavirus circulation cannot be completely eliminated. Therefore, sustained One Health-based surveillance integrating human cases, rodent hosts, and environmental information remains essential for early risk identification and effective prevention of HFRS [9,12].

Annual integrated surveillance remains necessary to characterize residual HFRS transmission and to identify priority areas, seasons, populations, and host-related risk signals. In 2025, Zhejiang Province continued routine human HFRS surveillance and rodent host monitoring. This study analyzed reported human HFRS cases and rodent surveillance data from Zhejiang Province in 2025. We aimed to describe the temporal, spatial, demographic, and diagnostic characteristics of human HFRS cases; summarize rodent density, species composition, and hantavirus RNA and antibody positivity; and assess the spatial correspondence between human case occurrence and rodent hantavirus surveillance signals. These findings are expected to provide evidence for targeted HFRS surveillance, early clinical recognition, vaccination strategies, and rodent control in low-incidence settings.

## 2. Materials and Methods

### 2.1. Data Sources

Data on reported human HFRS cases in Zhejiang Province in 2025 were obtained from the National Notifiable Disease Surveillance System (NNDSS). Cases were summarized by date of symptom onset and residential address. Population data were obtained from the Zhejiang Statistical Yearbook and were used to calculate incidence rates. County-level administrative boundary data were used for spatial mapping. Rodent surveillance data were collected from routine HFRS monitoring sites and included rodent density, species composition, hantavirus detection in lung specimens, and hantavirus antibody detection in serum specimens.

### 2.2. Case Definitions and Epidemiological Indicators

Reported HFRS cases were diagnosed according to the national diagnostic criteria for HFRS in China (WS 278–2008). According to the criteria, a case was defined as a patient presenting with corresponding clinical manifestations (fever, hemorrhage, renal dysfunction, and/or hypotension) and confirmed by any of the following laboratory methods: (1) detection of hantavirus-specific IgM antibodies in serum; (2) a ≥4-fold increase in hantavirus-specific IgG antibody titers between acute and convalescent sera; (3) detection of hantaviral RNA or antibody in clinical specimens; or (4) isolation of hantavirus from patient specimens. Incidence was calculated as the number of reported cases divided by the corresponding resident population and expressed per 100,000 population. Demographic characteristics were summarized by sex, age group, and occupation. The diagnostic interval was defined as the number of days between symptom onset and diagnosis and was used to assess diagnostic timeliness. Incidence was calculated as the number of reported cases divided by the corresponding resident population and expressed per 100,000 population. Demographic characteristics were summarized by sex, age group, and occupation. The diagnostic interval was defined as the number of days between symptom onset and diagnosis and was used to assess diagnostic timeliness.

### 2.3. Small-Mammal Host Surveillance

Small-mammal surveillance was conducted at five designated HFRS monitoring sites in Zhejiang Province in 2025, including Tiantai County, Longyou County, Lishui City, Ninghai County, and Jiangshan City. Trapping was performed in April and September in Tiantai County and in September at the other four sites, with approximately 100 small mammals collected per survey site. The September sampling was conducted approximately one month before the expected seasonal peak of HFRS occurrence in Zhejiang Province to assess rodent density and hantavirus circulation prior to the high-risk transmission period.

Outdoor trapping was performed in field environments using a 5 m trap-line method. Traps baited with peanuts were placed in the late afternoon and collected the following morning for three consecutive nights. Indoor trapping was conducted in residential areas, with at least 20 households selected at each site and five traps placed in each household for three consecutive nights. At least 100 trap-nights were included per day at each surveillance site. Captured rodents and shrews were identified to the species level based on morphological characteristics. Rodent density was calculated as the number of captured animals per 100 valid trap-nights.

### 2.4. Sample Collection and Laboratory Testing

Lung tissues and blood samples were collected from captured small mammals, and all samples were stored at −80 °C until further analysis.

Serological testing (indirect immunofluorescence assay, IFA): Hantavirus-specific antibodies in serum samples were detected using an indirect immunofluorescence assay (IFA). HFRS-related immunofluorescence antigen slides provided by the HFRS Laboratory of the Zhejiang Provincial Center for Disease Control and Prevention were used as the antigen substrate, and fluorescein-conjugated rabbit anti-mouse immunoglobulin (Sigma-Aldrich, St. Louis, MO, USA) was used as the secondary antibody.

Molecular detection (real-time RT-PCR): Total RNA was extracted from lung tissues using the GeneReady PI Animal Sample Pretreatment Kit (Shuzeng Biotechnology Co., Ltd., Hangzhou, China) in combination with an automated cryogenic centrifugal grinding system (Daan Gene Co., Ltd., Guangzhou, China). Hantavirus RNA was detected using a universal real-time fluorescent RT-PCR kit for hemorrhagic fever with renal syndrome hantavirus based on the TaqMan probe method (product code A5841YH; Beijing Zhuocheng Huisheng Biotechnology Co., Ltd., Beijing, China). A sample was considered positive when a typical sigmoidal amplification curve was observed in the FAM channel with a Ct value ≤ 38. Samples with 38 < Ct < 40 were re-extracted and retested, whereas samples without a typical amplification curve or with Ct ≥ 40 were considered negative. Kit-provided positive and negative controls were included in each run.

### 2.5. Statistical Analysis

Surveillance data were checked, cleaned, and summarized using WPS Office spreadsheets. Statistical analyses were performed using R software (version 4.4.3). Categorical variables were summarized as counts and proportions, and incidence rates were calculated per 100,000 population. Continuous variables were assessed for distribution normality using the Shapiro–Wilk test. Normally distributed variables were summarized as mean ± standard deviation (SD), whereas non-normally distributed variables were summarized as median with interquartile range (IQR).

Descriptive epidemiological analyses were performed to characterize the temporal, spatial, demographic, and diagnostic characteristics of reported HFRS cases. Chi-square goodness-of-fit tests were used to evaluate differences in monthly, seasonal, and prefecture-level distributions of human cases. Exact binomial tests and Pearson chi-square tests were used to assess differences in sex distribution, age-group distribution, and diagnostic interval categories. Fisher’s exact test was applied when expected frequencies were small.

For small-mammal surveillance data, Pearson chi-square tests were used to compare differences in capture density, host species composition, and hantavirus positivity among surveillance sites. Species-specific positivity rates were calculated descriptively, and estimates from species with small sample sizes were interpreted cautiously.

County-level incidence maps and integrated surveillance maps were generated using ArcGIS 10.7 and R software (version 4.4.3). All statistical tests were two-sided, and *p* < 0.05 was considered statistically significant.

## 3. Results

### 3.1. Human HFRS Cases and Temporal Distribution

**Figure 1:**
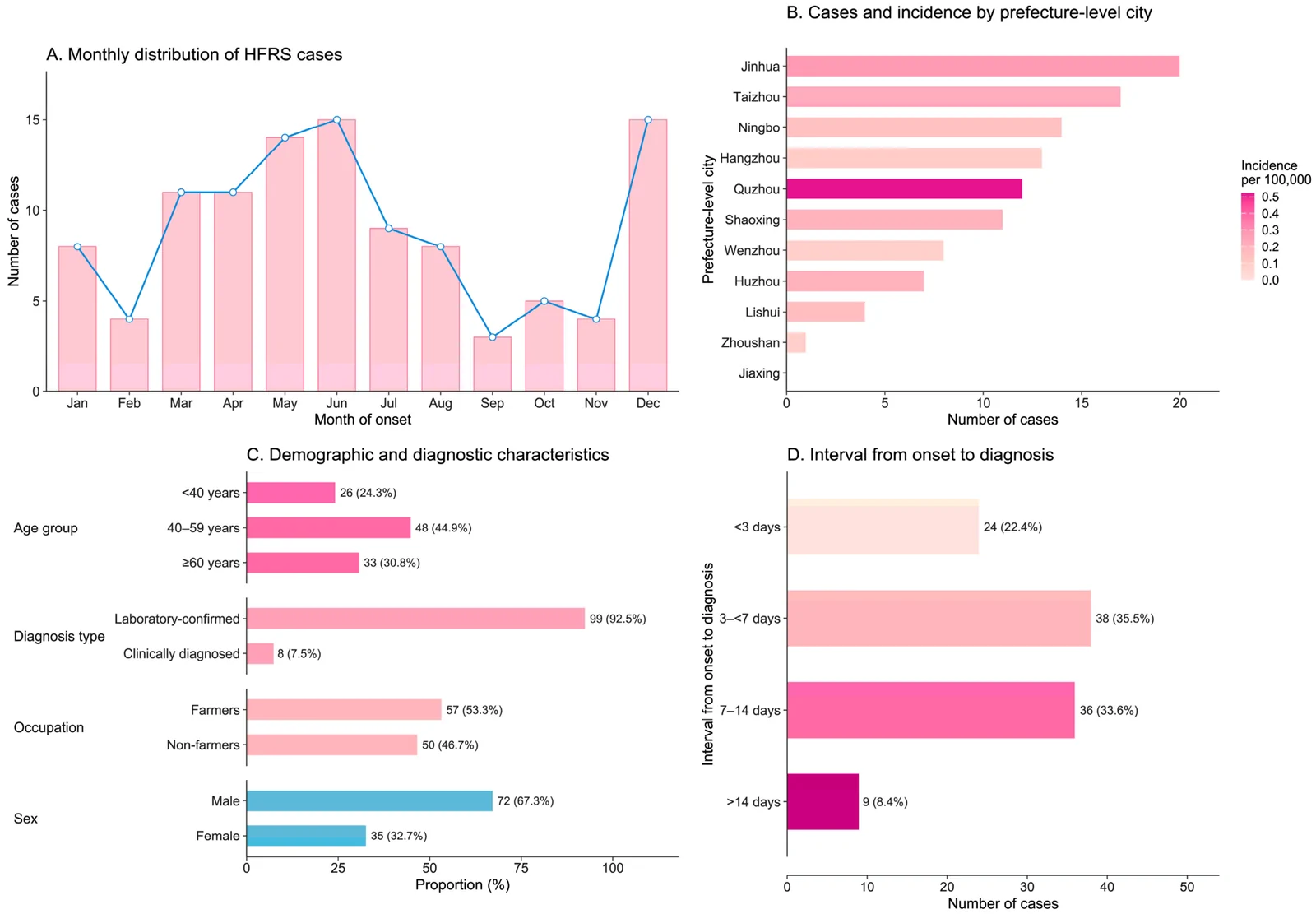
Epidemiological characteristics of reported HFRS cases in Zhejiang Province, China, 2025. (**A**) Monthly distribution of cases by symptom onset. (**B**) Case counts and incidence by prefecture-level city. Cities are ranked by case count, and bar color indicates incidence per 100,000 population. (**C**) Distribution of cases by age group, diagnosis type, occupation, and sex. (**D**) Interval from symptom onset to diagnosis.

In 2025, a total of 107 HFRS cases were reported in Zhejiang Province, including 99 laboratory-confirmed cases (92.5%) and 8 clinically diagnosed cases (7.5%). Cases occurred throughout the year, with monthly counts ranging from 3 to 15 cases (Figure 1A). The monthly distribution showed significant heterogeneity (χ^2^ = 23.43, df = 11, *p* = 0.015), with two prominent peaks observed in June and December (both *n* = 15).

Seasonally, cases were most frequently reported in spring (March–May, 36 cases, 33.6%), followed by summer (June–August, 32 cases, 29.9%), winter (December–February, 27 cases, 25.2%), and autumn (September–November, 12 cases, 11.2%). The seasonal distribution differed significantly from a uniform distribution (χ^2^ = 12.36, df = 3, *p* = 0.006).

### 3.2. Spatial Distribution of Human HFRS Cases

HFRS cases were reported in 10 of the 11 prefecture-level cities in Zhejiang Province, with no cases recorded in Jiaxing (Figure 1B). The distribution of cases among prefectures was significantly heterogeneous (χ^2^ = 45.48, df = 10, *p* < 0.001). Jinhua reported the highest number of cases (20/107, 18.7%), followed by Taizhou (17, 15.9%), Ningbo (14, 13.1%), Hangzhou (13, 12.1%), and Quzhou (12, 11.2%).

**Figure 2:**
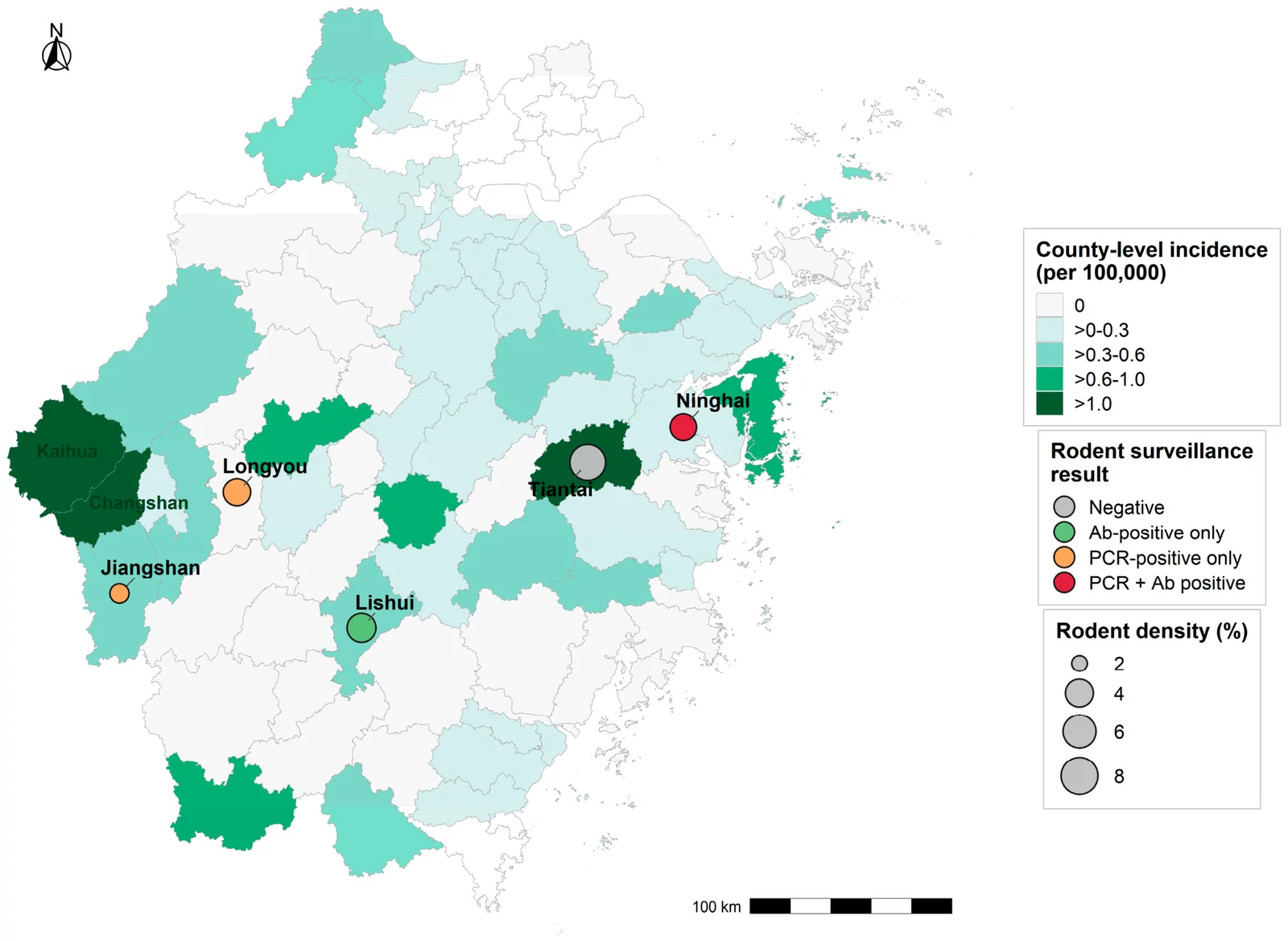
Spatial distribution of human HFRS incidence and rodent hantavirus surveillance signals in Zhejiang Province, China, 2025. County-level fill colors indicate the incidence of reported human HFRS cases per 100,000 population. Circles indicate rodent surveillance sites, with circle size proportional to rodent density and circle color representing hantavirus detection results in captured animals. Grey indicates no hantavirus RNA or antibody positivity, green indicates Ab-positive only, orange indicates PCR-positive only, and red indicates both PCR and Ab positivity. PCR, real-time reverse transcription polymerase chain reaction; Ab, antibody.

However, the geographic pattern differed when population size was considered. Quzhou showed the highest incidence (0.522 per 100,000), followed by Jinhua (0.277 per 100,000) and Taizhou (0.253 per 100,000), indicating that areas with high case numbers were not necessarily those with the highest population-adjusted risk. At the county level, cases occurred in 46 of the 90 counties, cities, or districts (Figure 2). Most affected counties had relatively low incidence levels (>0–0.3 per 100,000, *n* = 27), whereas three counties showed incidence exceeding 1.0 per 100,000: Tiantai (1.709 per 100,000), Kaihua (1.633 per 100,000), and Changshan (1.136 per 100,000). These counties represent priority areas for targeted surveillance and prevention.

### 3.3. Demographic and Diagnostic Characteristics

Reported HFRS cases were predominantly male. Among the 107 cases, 72 were male (67.3%), and 35 were female (32.7%), resulting in a male-to-female ratio of 2.06:1 (Figure 1C). The sex distribution differed significantly from an equal distribution (exact binomial test, *p* < 0.001).

Cases were mainly concentrated among middle-aged and older adults. Individuals aged 40–59 years accounted for the largest proportion of cases (48/107, 44.9%), followed by those aged ≥60 years (33/107, 30.8%) and those aged <40 years (26/107, 24.3%). The age distribution was significantly uneven (χ^2^ = 7.09, df = 2, *p* = 0.029), with 81 cases (75.7%) occurring among individuals aged ≥40 years.

By occupation, farmers accounted for the largest proportion of cases (57/107, 53.3%), followed by non-farmers (50/107, 46.7%).

The interval between symptom onset and diagnosis was mainly concentrated within 3–14 days (Figure 1D). Specifically, 24 cases (22.4%) were diagnosed within 3 days after symptom onset, 38 cases (35.5%) within 3 to <7 days, 36 cases (33.6%) within 7–14 days, and 9 cases (8.4%) more than 14 days after symptom onset. The distribution of diagnostic intervals differed significantly (χ^2^ = 21.77, df = 3, *p* < 0.001), with 45 cases (42.1%) diagnosed ≥7 days after symptom onset.

The demographic analyses were based on the composition of reported cases, as population denominators stratified by sex, age, and occupation were unavailable in the surveillance dataset.

### 3.4. Small-Mammal Surveillance and Hantavirus Positivity

Rodent host surveillance was conducted at five designated HFRS surveillance sites in Zhejiang Province, including the national surveillance site in Tiantai County and four provincial surveillance sites in Longyou County, Ninghai County, Jiangshan City, and Lishui City.

A total of 15,457 valid trap-nights were recorded, and 617 rodents and shrews were captured, corresponding to an overall capture density of 3.99% (Table 1, Figure 2). Capture density differed significantly among surveillance sites (χ^2^ = 119.85, df = 4, *p* < 0.001), with the highest density observed in Tiantai County (7.25%), followed by Lishui (4.36%), Longyou (3.84%), Ninghai (3.57%), and Jiangshan (2.17%). Eleven small-mammal species were identified, with *Apodemus agrarius* (187/617, 30.3%) and *Rattus norvegicus* (185/617, 30.0%) being the predominant species and accounting for 60.3% of all captured animals. Species composition varied significantly across surveillance sites (χ^2^ = 873.85, df = 40, *p* < 0.001), with different dominant species observed among sites (Supplementary Table S1).

**Figure 3:**
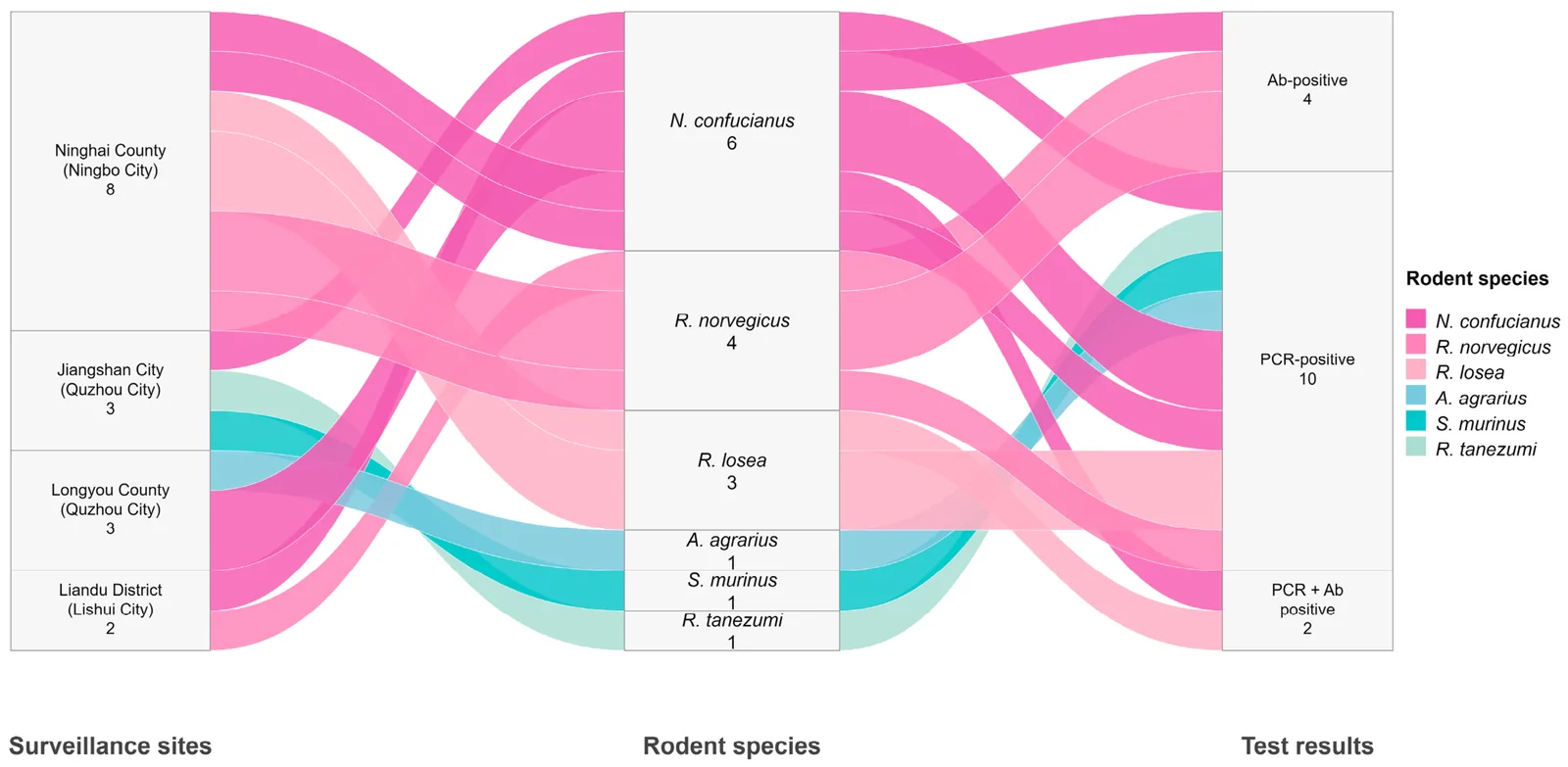
Sources of hantavirus-positive rodents and shrews in Zhejiang Province, China, 2025. The Sankey diagram shows the distribution of animals positive for hantavirus PCR and/or antibody by surveillance site, host species, and laboratory result category. Only hantavirus-positive animals are shown (n = 16). Flow width represents the number of positive animals. Species names are abbreviated as follows: *N. confucianus*, *Niviventer confucianus*; *R. norvegicus*, *Rattus norvegicus*; *R. losea*, *Rattus losea*; *A. agrarius*, *Apodemus agrarius*; *S. murinus*, *Suncus murinus*; *R. tanezumi*, *Rattus tanezumi*.

Among the 617 captured small mammals, 16 were positive for hantavirus RNA and/or antibody, resulting in an overall positivity rate of 2.59% (Table 1; Figure 2). Of these, 10 were PCR-positive only, 4 were antibody-positive only, and 2 were positive for both PCR and Ab. Hantavirus RNA was detected by real-time RT-PCR in 12 lung specimens (1.94%), whereas antibody positivity was detected in 6 serum samples (0.97%). The distribution of PCR positivity, antibody positivity, and overall positivity differed significantly among surveillance sites (all *p* < 0.01). Ninghai County showed the highest number of positive animals (8/103, 7.8%), whereas no positive animals were detected in Tiantai County despite its highest capture density. Longyou and Jiangshan showed PCR-positive animals only, while Lishui showed antibody-positive animals only (Table 1, Figure 3).

At the species level, hantavirus positivity was detected in six species, including *Niviventer confucianus* (6/51, 11.8%), *Rattus norvegicus* (4/185, 2.2%), *Rattus losea* (3/47, 6.4%), *Apodemus agrarius* (1/187, 0.5%), *Suncus murinus* (1/51, 2.0%), and *Rattus tanezumi* (1/46, 2.2%) (Supplementary Table S1). Species-level differences in positivity were statistically significant (χ^2^ = 24.37, df = 6, *p* < 0.001), although estimates for species with small sample sizes should be interpreted cautiously. The Sankey diagram further illustrated that hantavirus-positive animals were concentrated in specific surveillance sites and host species rather than being evenly distributed across the surveillance network (Figure 3).

### 3.5. Integrated Interpretation of Human Cases and Rodent Surveillance Signals

The integrated analysis combining county-level human HFRS incidence and small-mammal surveillance data revealed heterogeneous patterns of human cases and hantavirus detection across surveillance sites (Figure 2). The highest county-level human incidence was observed in Tiantai County, Kaihua County, and Changshan County. Among the five small-mammal surveillance sites, hantavirus detection was observed in Ninghai, Longyou, Jiangshan, and Lishui, whereas no positive animals were detected in Tiantai County despite its highest capture density.

The spatial patterns of human incidence, small-mammal capture density, host species composition, and hantavirus positivity were not fully concordant. Tiantai County showed both the highest human incidence and the highest capture density but no detectable hantavirus markers in sampled animals. In contrast, Ninghai County showed both PCR and antibody positivity among small mammals, although its human incidence was lower than that of the highest-incidence counties. These findings demonstrate differences between human surveillance outcomes and host-based surveillance signals across local settings.

The Sankey diagram illustrated the distribution of hantavirus-positive animals by surveillance site, host species, and laboratory detection category (Figure 3). Among the 16 positive animals, Ninghai County contributed the largest number (*n* = 8), followed by Longyou County (*n* = 3), Jiangshan City (*n* = 3), and Lishui City (*n* = 2), whereas no positive animals were detected in Tiantai County. Positive animals were identified in six species, with Niviventer confucianus accounting for the highest number (*n* = 6), followed by *Rattus norvegicus* (*n* = 4) and *Rattus losea* (*n* = 3). According to laboratory detection categories, 10 animals were PCR-positive only, 4 were antibody-positive only, and 2 were positive for both markers.

Overall, hantavirus-positive animals were concentrated in specific surveillance sites and host species rather than being evenly distributed across the surveillance network. Ninghai County was the only site showing both PCR and antibody positivity, with positive animals mainly detected in *R. losea*, *R. norvegicus*, and *N. confucianus*. Longyou and Jiangshan contributed PCR-positive animals only, whereas Lishui contributed antibody-positive animals only.

## 4. Discussion

This study provides an integrated assessment of human HFRS cases and rodent hantavirus surveillance in Zhejiang Province, China, in 2025. Although the overall number of reported cases was low, HFRS continued to occur throughout the year and showed clear spatial heterogeneity. Human cases were concentrated among males, middle-aged and older adults, and farmers, indicating that occupational and environmental exposure remains important in the current low-incidence stage [8]. Small-mammal surveillance detected hantavirus RNA and/or antibody signals in several surveillance sites and host species, indicating evidence of hantavirus circulation among the sampled small mammals [13]. These findings indicate that residual HFRS risk in Zhejiang should be interpreted through both human case occurrence and rodent-host surveillance signals [1,6].

Human cases showed a bimodal seasonal pattern, with peaks in June and December. The early-summer peak may be related to increased agricultural activity and outdoor exposure to rodent-contaminated environments, while the winter peak is consistent with the traditional cold-season increase observed in many HFRS-endemic areas [9,14]. However, because this analysis was based on a single surveillance year, the seasonal pattern should be interpreted as a descriptive signal rather than a stable long-term trend. Continued multi-year surveillance is required to determine whether this pattern persists [15,16].

The spatial distribution of HFRS cases was heterogeneous. Jinhua reported the largest number of cases, whereas Quzhou had the highest prefecture-level incidence. At the county level, Tiantai, Kaihua, and Changshan had the highest incidence. This distinction between absolute case counts and population-adjusted incidence is important for surveillance prioritization. In low-incidence settings, large cities may contribute more cases because of population size, while smaller counties may have higher incidence and greater local risk [17]. Therefore, both case numbers and incidence should be considered when identifying priority areas for clinical preparedness, vaccination, environmental management, and rodent control [18].

The demographic pattern was consistent with the expected exposure pathways of rodent-borne hantavirus infection. Males, people aged 40 years or older, and farmers accounted for most reported cases. These groups may have more frequent contact with rodent habitats, agricultural fields, grain storage areas, residential surroundings, and other peri-domestic environments [19]. Importantly, the population groups affected by HFRS may change as landscapes become urbanized. The Xi’an study reported that urbanization had different effects on farmers and non-farmers, implying that urban expansion may modify human exposure pathways rather than simply reducing traditional rural risk [20]. Health education, vaccination promotion among eligible high-risk groups, environmental sanitation, rodent-proofing, and reduction in rodent contamination in household and agricultural settings remain important [21].

In our study, diagnostic delay was observed in some patients, particularly during periods of low disease incidence. This finding highlights the challenge of early clinical recognition of HFRS in routine practice. Previous studies have reported that scrub typhus and HFRS may share overlapping clinical manifestations and that misdiagnosis between these two diseases can occur, emphasizing the importance of laboratory confirmation for differential diagnosis [22]. Because early HFRS symptoms are non-specific and may resemble other acute febrile illnesses, delayed diagnosis may occur when clinical suspicion is low, especially in areas with sporadic or historically low incidence. The high proportion of laboratory-confirmed cases in this study indicates that diagnostic testing remains central to case confirmation [8,23]. Maintaining clinician awareness and access to laboratory testing in both high-incidence and lower-incidence areas is essential for early recognition, patient management, and surveillance sensitivity [11,24,25].

Small-mammal surveillance provides important evidence for assessing the potential spillover risk of hantaviruses from animal hosts to humans. Among the 617 captured rodents and shrews, 16 animals were positive for hantavirus, indicating that hantavirus infection or exposure was present in local small-mammal communities. Although these findings cannot directly link specific positive animals to human HFRS cases, they suggest that infected or previously exposed hosts were active in environments where human contact may occur. Hantavirus RNA positivity indicates the detection of viral genetic material at the time of sampling, whereas antibody positivity reflects host immune responses following exposure [26]. Therefore, these two indicators provide complementary but not identical information on hantavirus circulation. RNA and antibody detection results should be interpreted together as complementary signals of enzootic circulation and potential human exposure risk [27].

The host species’ findings further highlight the importance of the human–rodent interface. Hantavirus-positive animals were detected in multiple species, including *Niviventer confucianus*, *Rattus norvegicus*, *Rattus losea*, *Apodemus agrarius*, *Suncus murinus*, and *Rattus tanezumi*, suggesting that potential exposure risk may arise from a broader host community rather than from a single dominant species. This is particularly relevant for species associated with residential, peri-domestic, agricultural, and field–village environments.

Commensal rats such as *R. norvegicus* and *R. tanezumi* may contaminate household surroundings, storage areas, livestock or poultry sheds, and village environments, whereas field-associated species such as *R. losea* and *A. agrarius* may increase exposure during agricultural activities [23,25].

Although *S. murinus* is a shrew rather than a classical rodent host of HFRS-associated hantaviruses, its presence near residential environments may indicate broader zoonotic risk in small-mammal communities [28,29,30].

The integrated map and Sankey diagram showed that human incidence, rodent density, host species composition, and hantavirus positivity were not completely concordant. Tiantai County had the highest human incidence and rodent density but no hantavirus-positive animals in the available specimens, whereas Ninghai County showed both PCR and antibody positivity in rodents despite lower human incidence. This discordance may reflect differences in sampling timing, trapping habitat, host composition, sample size, local virus circulation, and laboratory marker type [31]. In addition, human mobility and occupational activities may contribute to spatial discrepancies between infection locations and residential locations. Individuals, particularly agricultural workers, may commute between villages, farmland, workplaces, and other environments where rodent exposure occurs; therefore, human infections may not always correspond geographically to the locations where infected rodents are detected [19]. Therefore, human case numbers, rodent density, and individual host detections should be interpreted together rather than used individually as indicators of local HFRS risk. Prevention should focus on contact zones where infected hosts, contaminated environments, and susceptible humans overlap, including residential compounds, storage rooms, livestock or poultry sheds, village margins, and agricultural fields [32,33].

HFRS represents a typical One Health disease, as its transmission is shaped by interactions among humans, rodent reservoirs, hantaviruses, and environmental conditions. According to the One Health framework, emerging and re-emerging zoonotic diseases are influenced by multiple ecological and anthropogenic drivers, including climate conditions, land-use changes, agricultural activities, and human–animal interactions [34]. Therefore, occupational characteristics should be interpreted as indicators of potential exposure scenarios rather than isolated risk factors. In Zhejiang Province, although long-term surveillance, rodent control, environmental improvement, and public health interventions have contributed to the sustained reduction in HFRS incidence, the persistence of natural reservoir habitats means that transmission risk cannot be completely eliminated. Continuous integration of human surveillance, rodent monitoring, pathogen characterization, and environmental information is essential for sustainable HFRS prevention and control [35].

From a prevention perspective, the key risk points are the contact zones where infected hosts, contaminated environments, and susceptible humans overlap. Residential compounds, storage rooms, livestock or poultry sheds, village margins, agricultural fields, and field–village transition zones should be considered priority settings for integrated risk reduction. In these settings, commensal rats, field rodents, shrews, and humans may share the same environment. Targeted rodent control, environmental sanitation, rodent-proof food storage, protection during fieldwork, and timely laboratory testing for febrile patients with a possible exposure history should be strengthened [36,37].

This study has several limitations. First, this analysis was based on one year of integrated human and rodent surveillance data and was primarily descriptive; therefore, causal relationships between rodent hantavirus circulation and human HFRS occurrence could not be established. Second, small-mammal surveillance was conducted at five designated monitoring sites rather than across all counties with reported human cases, and the spatial concordance between rodent infection signals and human infections should therefore be interpreted cautiously. Third, hantavirus infection and exposure in rodents were assessed by real-time RT-PCR detection of viral RNA in lung tissues and IFA detection of specific antibodies in serum samples. Although these approaches are widely used in hantavirus surveillance, they cannot determine viral genotypes or establish direct transmission links between rodents and humans. Future genomic sequencing and phylogenetic analyses are needed to characterize hantavirus diversity and transmission dynamics. Fourth, some rodent species had limited sample sizes, restricting species-specific comparisons of infection prevalence. Finally, environmental and individual-level risk factors, including climatic variables, land-use characteristics, agricultural activities, habitat features, vaccination status, and exposure history, were not incorporated into the current analysis. Future longitudinal studies integrating epidemiological, ecological, environmental, and molecular data within a One Health framework are needed to better characterize hantavirus transmission dynamics and improve risk prediction and prevention strategies.

Despite these limitations, this study demonstrates the practical value of combining routine human surveillance with active rodent host monitoring. In a low-incidence setting, sporadic human cases may still occur, and hantavirus activity may be detected in selected host species and human–rodent contact environments. Annual integrated surveillance can help identify priority areas and risk interfaces for clinical awareness, laboratory testing, vaccination, environmental management, and rodent control. Future surveillance should incorporate molecular detection, virus sequencing, and phylogenetic characterization to clarify circulating hantavirus types and strengthen the evidence base for targeted HFRS.

## 5. Conclusions

HFRS remained at a low but persistent level in Zhejiang Province in 2025, with clear temporal, spatial, and demographic heterogeneity. Rodent surveillance detected hantavirus PCR and/or antibody positivity in several host species, including species associated with residential, peri-domestic, and agricultural environments. Human incidence, rodent density, host composition, and hantavirus positivity were not fully concordant, indicating that HFRS risk assessment should integrate multiple surveillance signals rather than rely on any single indicator. Continued human–rodent integrated surveillance, combined with targeted environmental interventions and future molecular characterization of circulating hantaviruses, is needed to support early warning and precise prevention in low-incidence settings.

## Figures and Tables

**Table 1:** Rodent density, dominant species, and hantavirus detection results at surveillance sites in Zhejiang Province, China, 2025.

Surveillance Site	Valid Trap-Nights	Captured Animals, *n*	Rodent Density (%)	Dominant Species	PCR-Positive, *n* (%)	Ab-Positive, *n* (%)	Total Positive, *n* (%)
Longyou County	2841	109	3.84	*A. agrarius* (62), *N. confucianus* (15), *R. tanezumi* (13)	3 (2.75)	0 (0.00)	3 (2.75)
Jiangshan City	4600	100	2.17	*S. murinus* (50), *R. tanezumi* (20), M. musculus (16)	3 (3.00)	0 (0.00)	3 (3.00)
Lishui City	2315	101	4.36	*R. norvegicus* (73), *N. confucianus* (20), *E. melanogaster* (6)	0 (0.00)	2 (1.98)	2 (1.98)
Ninghai County	2886	103	3.57	*R. losea* (44), *R. norvegicus* (20), *A. agrarius* (12)	6 (5.83)	4 (3.88)	8 (7.77)
Tiantai County	2815	204	7.25	*A. agrarius* (109), *R. norvegicus* (74), *E. melanogaster* (14)	0 (0.00)	0 (0.00)	0 (0.00)
Total	15,457	617	3.99	*A. agrarius* (187), *R. norvegicus* (185), *S. murinus* and *N. confucianus* (51 each)	12 (1.94)	6 (0.97)	16 (2.59)

## Data Availability

The data presented in this study are available upon request from the corresponding author. The requirement for informed consent was waived because this study used deidentified routine surveillance data. All individual-level data were kept confidential.

## Supplementary Materials

The following supporting information can be downloaded at: https://www.mdpi.com/article/10.3390/v18090945/s1, Table S1: Species composition and hantavirus-positive rodents by surveillance site in Zhejiang Province, 2025.
