# Supplementary material for: Integrated Analysis of Human Hemorrhagic Fever with Renal Syndrome Cases and Rodent Hantavirus Surveillance in Zhejiang Province, China, 2025
Source: Viruses. 2026 Aug 28;18(9):945. doi: 10.3390/v18090945 (PMC13611933; doi:10.3390/v18090945)
Supplement: Supplementary file 1 [file viruses-18-00945-s001.zip › viruses-4451078-supplementary.pdf]

Supplementary Table S1. Species composition and hantavirus-positive rodents by surveillance site in Zhejiang Province, 2025

Surveillance site	Rodent	Captured, n	Proportion within site (%)	Ag-positive only, n	Ab-positive only, n	Ag + Ab positive, n	Total positive, n	Positivity among captured (%)
Longyou County	Apodemus agrarius	62	56.9	1	0	0	1	1.61
Longyou County	Niviventer confucianus	15	13.8	2	0	0	2	13.33
Longyou County	Rattus tanezumi	13	11.9	0	0	0	0	0.00
Longyou County	Rattus norvegicus	12	11.0	0	0	0	0	0.00
Longyou County	Berylmys bowersi	2	1.8	0	0	0	0	0.00
Longyou County	Eothenomys melanogaster	2	1.8	0	0	0	0	0.00
Longyou County	Mus musculus	2	1.8	0	0	0	0	0.00
Longyou County	Suncus murinus	1	0.9	0	0	0	0	0.00
**Longyou County**	**—**	**109**	**100.0**	**3**	**0**	**0**	**3**	**2.75**
Jiangshan City	Suncus murinus	50	50.0	1	0	0	1	2.00
Jiangshan City	Rattus tanezumi	20	20.0	1	0	0	1	5.00
Jiangshan City	Mus musculus	16	16.0	0	0	0	0	0.00
Jiangshan City	Rattus norvegicus	6	6.0	0	0	0	0	0.00
Jiangshan City	Apodemus agrarius	3	3.0	0	0	0	0	0.00
Jiangshan City	Rattus losea	3	3.0	0	0	0	0	0.00
Jiangshan City	Niviventer confucianus	2	2.0	1	0	0	1	50.00
**Jiangshan City**	**—**	**100**	**100.0**	**3**	**0**	**0**	**3**	**3.00**
Lishui City	Rattus norvegicus	73	72.3	0	1	0	1	1.37

Lishui City	Niviventer confucianus	20	19.8	0	1	0	1	5.00
Lishui City	Eothenomys melanogaster	6	5.9	0	0	0	0	0.00
Lishui City	Apodemus agrarius	1	1.0	0	0	0	0	0.00
Lishui City	Rattus tanezumi	1	1.0	0	0	0	0	0.00
**Lishui City**	**—**	**101**	**100.0**	**0**	**2**	**0**	**2**	**1.98**
Ninghai County	Rattus losea	44	42.7	2	0	1	3	6.82
Ninghai County	Rattus norvegicus	20	19.4	1	2	0	3	15.00
Ninghai County	Apodemus agrarius	12	11.7	0	0	0	0	0.00
Ninghai County	Niviventer confucianus	12	11.7	1	0	1	2	16.67
Ninghai County	Rattus tanezumi	10	9.7	0	0	0	0	0.00
Ninghai County	Leopoldamys edwardsi	5	4.9	0	0	0	0	0.00
**Ninghai County**	**—**	**103**	**100.0**	**4**	**2**	**2**	**8**	**7.77**
Tiantai County	Apodemus agrarius	109	53.4	0	0	0	0	0.00
Tiantai County	Rattus norvegicus	74	36.3	0	0	0	0	0.00
Tiantai County	Eothenomys melanogaster	14	6.9	0	0	0	0	0.00
Tiantai County	Leopoldamys edwardsi	2	1.0	0	0	0	0	0.00
Tiantai County	Niviventer confucianus	2	1.0	0	0	0	0	0.00
Tiantai County	Rattus tanezumi	2	1.0	0	0	0	0	0.00

Tiantai County	Rattus nitidus	1	0.5	0	0	0	0	0.00
**Tiantai County**	**—**	**204**	**100.0**	**0**	**0**	**0**	**0**	**0.00**
**Total**	**—**	**617**	**100.0**	**10**	**4**	**2**	**16**	**2.59**

Note: Ag, antigen; Ab, antibody. "Ag + Ab positive" indicates rodents positive for both hantavirus antigen and antibody.
